# Supplementary material for: Temporal matching as an accounting principle for green electricity claims
Source: Nat Commun. 2025 Oct 20;16:9280. doi: 10.1038/s41467-025-65125-z (PMC12537979; doi:10.1038/s41467-025-65125-z)
Supplement: Supplementary file 1 — Supplementary Information File [file 41467_2025_65125_MOESM1_ESM.pdf]

Supplementary information for temporal matching as an accounting principle for green electricity claims

Hanna F. Scholta[1*] and Maximilian J. Blaschke[1,2]

[1]Chair of Management Accounting, TUM School of Management,, Technische Universität München, Germany.
[2]Center for Energy and Environmental Policy Research, Massachusetts Institute of Technology, Cambridge, MA, USA.

*Corresponding author(s). E-mail(s): hanna.scholta@tum.de;

Supplementary Items List

Supplementary Figures..p.3

Supplementary figure 1...p.3

Supplementary figure 2...p.4

Supplementary figure 3...p.5

Supplementary Tables..p.6

Supplementary table 1...p.6

Supplementary table 2...p.6

Supplementary Note: Voluntary and mandatory green electricity markets................p.7

Supplementary References..p.8

Supplementary Figures

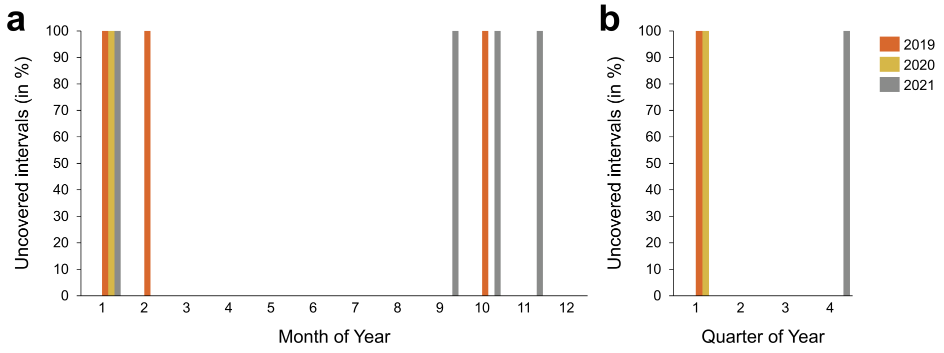

Supplementary Figure 1: **Distribution of uncovered intervals under quarterly and monthly matching, 2016-2021.** This figure shows the distribution of intervals in which green electricity demand was uncovered by green electricity supply under monthly (a) and quarterly (b) matching. While no uncovered intervals appear under either matching scheme from 2016 to 2018, shortages arise from 2019 (orange bars, the year 2020 is indicated by the yellow bars, the year 2021 by the grey bars) onwards. Under quarterly matching, they occur in either the first or fourth quarter. Monthly matching shows that shortages are concentrated in January, February, September, October, and November.

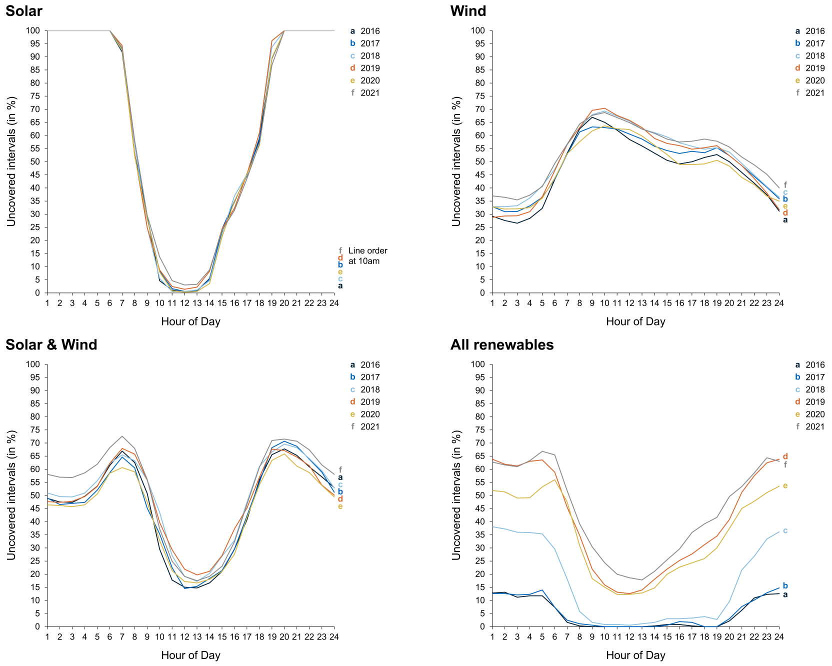

Supplementary Figure 2: **Distribution of hours with a shortage in green electricity supply over the day for hypothetical solar, wind, and hybrid-systems scenarios and our base case scenario across the years 2016-2021.** This figure highlights the role of the increase of variable renewable energy, in particular wind and solar, in the increase of day-night disparities through putting three hypothetical scenarios: solar-only electricity generation (panel a), wind-only electricity generation (panel b), and hybrid electricity generation (panel c), in comparison to the base case that we study in our paper (panel d). It shows the distribution of uncovered intervals across the day. Each year is displayed as a separate line (a: 2016, b: 2017, c: 2018, d: 2019, e: 2020, f: 2021). The day-night disparity climaxes in a system powered exclusively by solar energy, with the proportion of uncovered intervals rising to 100% during nighttime. The surplus generated during the daytime, however, sufficiently offsets the shortages of the night on an annually aggregated level. Different dynamics are at play in a system relying solely on wind energy. The incidence of uncovered intervals at night is notably lower. Instead, we see a tendency for shortages to peak in the morning. Interestingly, a hybrid system that integrates both solar and wind resources does not eliminate the day-night dichotomy. Instead, it moderates the extremities, curtailing the frequency of uncovered intervals at night at the expense of an increase during the day. If we view the base case against the hypothetical scenarios, the intensification of the share of uncovered intervals over the years appears to reflect a convergence towards the hypothetical hybrid system.

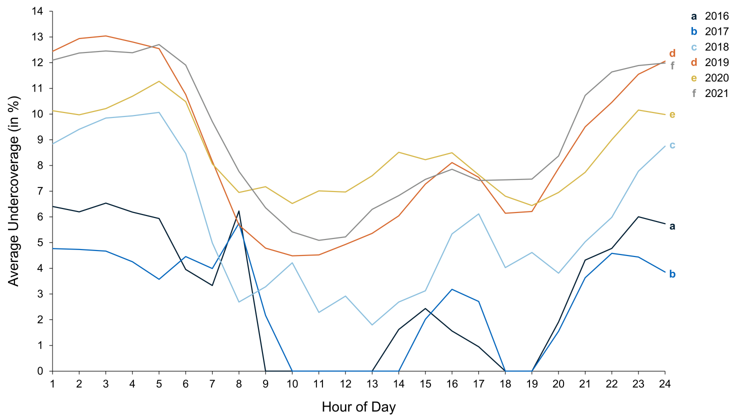

Supplementary Figure 3: **Volumetric scale of under-coverage during hours of green electricity supply shortages per year from 2016-2021.** This figure shows the average under-coverage of hours across the day that face green electricity supply shortages. The average shortage is displayed in percent of total GO Demand at the respective hour of day, and each year is represented by a separate line (a: 2016, b: 2017, c: 2018, d: 2019, e: 2020, f: 2021). Contrasting line a (2016) with line e (2020) and line f (2021) shows that the overall magnitude of under-coverage increases, as well as the increase in the disparity between day and night hours.

Supplementary Tables

Supplementary Table 1: Summed |Under-coverage| under each temporal matching requirement relative to yearly GO Demand (of all intervals) per year

Year	Quarterly	Monthly	Weekly	Daily	Hourly
2016	0	0	0	0	0.2
2017	0	0	0	0	0.2
2018	0	0	0.1	0.2	1.1
2019	0.4	1.4	1.6	2.7	3.8
2020	0.2	1.0	1.3	2.2	3.1
2021	0.7	1.4	2.5	3.4	4.3

Supplementary Table 2: Countries included in our analysis and the respective analysis periods, based on their connection to the AIB hub between 2016 and 2021

**No.**	**Country**	**From**	**Until**
1	Austria	2016	2021
2	Belgium	2016	2021
3	Switzerland	2016	2021
4	Cyprus	2016	2021
5	Czech Republic	2016	2021
6	Germany	2016	2021
7	Denmark	2016	2021
8	Estonia	2016	2021
9	Spain	2016	2021
10	Finland	2016	2021
11	France	2016	2021
12	Croatia	2016	2021
13	Ireland	2016	2021
14	Iceland	2016	2021
15	Italy	2016	2021
16	Luxembourg	2016	2021
17	Latvia	2018	2021
18	Netherlands	2016	2021
19	Norway	2016	2021
20	Portugal	2020	2021
21	Serbia	Oct 2019	2021
22	Sweden	2016	2021
23	Slovenia	2016	2021
24	Slovakia	Oct 2019	2021

Supplementary Note: Voluntary and mandatory green electricity markets

Demand for green electricity generally can arise in two forms (voluntary and mandatory), resulting in two different types of markets:

In voluntary markets, which we study in our paper, demand depends on the willingness of consumers or companies to make commitments beyond what is legally required. Voluntary consumption of green electricity can complement governmental support policies for renewable energy adoption, such as feed-in tariffs, tax credits, or usage mandates [1].

In mandatory markets, demand arises from government-enforced quota obligations (often referred to as renewable portfolio standards). Non-compliance with quotas typically results in penalty payments. Examples include the Swedish–Norwegian Tradable Green Certificate Market and Renewable Energy Certificate (REC) compliance markets in the United States.

Supplementary References

[1] Raadal, H. L., Dotzauer, E., Hanssen, O. J. & Kildal, H. P. The interaction between Electricity Disclosure and Tradable Green Certificates. *Energy Policy* **42**, 419–428 (2012).
