# Supplementary material for: Temporal matching as an accounting principle for green electricity claims
Source: Nat Commun. 2025 Oct 20;16:9280. doi: 10.1038/s41467-025-65125-z (PMC12537979; doi:10.1038/s41467-025-65125-z)
Supplement: Supplementary file 2 — Reporting Summary [file 41467_2025_65125_MOESM2_ESM.pdf]

nature portfolio

Corresponding author(s): Hanna F. Scholta

Last updated by author(s): Sep 11, 2025

Reporting Summary

Nature Portfolio wishes to improve the reproducibility of the work that we publish. This form provides structure for consistency and transparency in reporting. For further information on Nature Portfolio policies, see our Editorial Policies and the Editorial Policy Checklist.

Statistics

For all statistical analyses, confirm that the following items are present in the figure legend, table legend, main text, or Methods section.

n/a	Confirmed	
☒	☐	The exact sample size (*n*) for each experimental group/condition, given as a discrete number and unit of measurement
☒	☐	A statement on whether measurements were taken from distinct samples or whether the same sample was measured repeatedly
☒	☐	The statistical test(s) used AND whether they are one- or two-sided<br>*Only common tests should be described solely by name; describe more complex techniques in the Methods section.*
☒	☐	A description of all covariates tested
☒	☐	A description of any assumptions or corrections, such as tests of normality and adjustment for multiple comparisons
☐	☒	A full description of the statistical parameters including central tendency (e.g. means) or other basic estimates (e.g. regression coefficient) AND variation (e.g. standard deviation) or associated estimates of uncertainty (e.g. confidence intervals)
☒	☐	For null hypothesis testing, the test statistic (e.g. *F*, *t*, *r*) with confidence intervals, effect sizes, degrees of freedom and *P* value noted<br>*Give P values as exact values whenever suitable.*
☒	☐	For Bayesian analysis, information on the choice of priors and Markov chain Monte Carlo settings
☒	☐	For hierarchical and complex designs, identification of the appropriate level for tests and full reporting of outcomes
☒	☐	Estimates of effect sizes (e.g. Cohen's *d*, Pearson's *r*), indicating how they were calculated

Our web collection on statistics for biologists contains articles on many of the points above.

Software and code

Policy information about availability of computer code

Data collection	Data preprocessing was conducted in R (v4.3.0) using RStudio (v2023.12.1). Data preprocessing scripts are available on GitHub https://github.com/HannaFScholta/Temporal-matching-as-an-accounting-principle-for-green-electricity-claims. All code has been archived on Zenodo under https://doi.org/10.5281/zenodo.17198701
Data analysis	Data analysis was conducted using Microsoft Excel and R (v4.3.0) with RStudio (v2023.12.1). Analysis code is available on GitHub https://github.com/HannaFScholta/Temporal-matching-as-an-accounting-principle-for-green-electricity-claims, and has been archived on Zenodo under https://doi.org/10.5281/zenodo.17198701

For manuscripts utilizing custom algorithms or software that are central to the research but not yet described in published literature, software must be made available to editors and reviewers. We strongly encourage code deposition in a community repository (e.g. GitHub). See the Nature Portfolio guidelines for submitting code & software for further information.

Data

Policy information about availability of data

All manuscripts must include a data availability statement. This statement should provide the following information, where applicable:

- Accession codes, unique identifiers, or web links for publicly available datasets
- A description of any restrictions on data availability
- For clinical datasets or third party data, please ensure that the statement adheres to our policy

Source data are provided with this paper. The analysis data is available on GitHub https://github.com/HannaFScholta/Temporal-matching-as-an-accounting-principle-for-green-electricity-claims and has been archived, alongside the code, on Zenodo https://doi.org/10.5281/zenodo.17198701. The raw data used in this work is publicly available from the cited sources, which are also linked in the GitHub repository.

Research involving human participants, their data, or biological material

Policy information about studies with human participants or human data. See also policy information about sex, gender (identity/presentation), and sexual orientation and race, ethnicity and racism.

Reporting on sex and gender	n/a
Reporting on race, ethnicity, or other socially relevant groupings	n/a
Population characteristics	n/a
Recruitment	n/a
Ethics oversight	n/a

Note that full information on the approval of the study protocol must also be provided in the manuscript.

Field-specific reporting

Please select the one below that is the best fit for your research. If you are not sure, read the appropriate sections before making your selection.

☐ Life sciences ☐ Behavioural & social sciences ☒ Ecological, evolutionary & environmental sciences

For a reference copy of the document with all sections, see nature.com/documents/nr-reporting-summary-flat.pdf

Ecological, evolutionary & environmental sciences study design

All studies must disclose on these points even when the disclosure is negative.

Study description	This study combines historical certificate data with electricity market data to assess the implications of increasingly strict temporal matching requirements (quarterly, monthly, weekly, daily, hourly) on green electricity claims in the European Guarantees of Origin (GO) market from 2016 to 2021.
Research sample	Our analysis includes all countries connected via the so-called Association of Issuing Bodies (AIB) Hub - which is the hub that connects the GO market - from the years 2016 to 2021. Representing the GO market as a whole, the following countries are part of our analsis over the noted timeframe: Austria (2016–2021), Belgium (2016–2021), Switzerland (2016–2021), Cyprus (2016–2021), Czech Republic (2016–2021), Germany (2016–2021), Denmark (2016–2021), Estonia (2016–2021), Spain (2016–2021), Finland (2016–2021), France (2016–2021), Croatia (2016–2021), Ireland (2016–2021), Iceland (2016–2021), Italy (2016–2021), Luxembourg (2016–2021), Latvia (2018–2021), Netherlands (2016–2021), Norway (2016–2021), Portugal (2020–2021), Serbia (Oct 2019–2021), Sweden (2016–2021), Slovenia (2016–2021), Slovakia (Oct 2019–2021). The main data set used to derive certificate supply and demand was the AIB statistics (see Methods/GitHub repository).
Sampling strategy	The years 2016–2020 were selected to capture developments over the most recent five-year period with fully completed certificate cancellations. Since certificates can be cancelled for up to 18 months in retrospect, cancellations for certificates issued in December 2020 were possible until mid-2022, and data collection started early 2023 (see below). As cancellations for 2021 became finalized by mid-2023 while the analysis was still in progress, this year was also included for completeness. The data collection was closed thereafter. All 24 European countries connected via the AIB hub over the covered timeframe were considered in the analysis.
Data collection	All data used in this study were obtained from publicly available sources. Data from the European Network of Transmission System Operators for Electricity (entso-e) were accessed via API, while data from AIB, the Statistical Office of the European Union (Eurostat), and the International Energy Agency (IEA) were downloaded as Excel or CSV files from their respective websites (see Methods/GitHub repository). The AIB provides statistics on historical GO issuance, cancellation, and trade. Entso-e supplies up to quarter-

	hourly data on electricity generation and consumption within Europe. In addition, we obtained net electricity production data for Croatia from the IEA, and electricity consumption data for Cyprus from Eurostat.
Timing and spatial scale	Data were collected from publicly available AIB, entso-e, Eurostat, and IEA datasets between March 8, 2023, and July 5, 2023, spanning all countries connected via the AIB Hub between the years 2016 to 2021 (see "Research sample" for detailed description)
Data exclusions	No data points were excluded, except where input datasets contained missing values; these were handled using a structured data imputation approach (see Methods).
Reproducibility	All data sources are publicly available, and all code for data processing and analysis is openly available at: https://github.com/HannaFScholta/Temporal-matching-as-an-accounting-principle-for-green-electricity-claims
Randomization	n/a
Blinding	n/a

Did the study involve field work? ☐ Yes ☒ No

Reporting for specific materials, systems and methods

We require information from authors about some types of materials, experimental systems and methods used in many studies. Here, indicate whether each material, system or method listed is relevant to your study. If you are not sure if a list item applies to your research, read the appropriate section before selecting a response.

Materials & experimental systems

n/a	Involved in the study	
☒	☐	Antibodies
☒	☐	Eukaryotic cell lines
☒	☐	Palaeontology and archaeology
☒	☐	Animals and other organisms
☒	☐	Clinical data
☒	☐	Dual use research of concern
☒	☐	Plants

Methods

n/a	Involved in the study	
☒	☐	ChIP-seq
☒	☐	Flow cytometry
☒	☐	MRI-based neuroimaging

Plants

Seed stocks	n/a
Novel plant genotypes	n/a
Authentication	n/a
